# Supplementary material for: Integrated Microbiota and Metabolomics Analysis of Candida utilis CU-3 Solid-State Fermentation Effects on Cottonseed Hull-Based Feed
Source: Microorganisms. 2025 Jun 13;13(6):1380. doi: 10.3390/microorganisms13061380 (PMC12196524; doi:10.3390/microorganisms13061380)
Supplement: Supplementary file 1 [file microorganisms-13-01380-s001.zip › microorganisms-3638646-supplementary.pdf]

Table S1. Variation of nutrient species content in feed samples

ID	Name	Molecular Weigh	P-value	VIP	FC	Up.Down
**Positive ion mode**						
Com_987_pos	Cycloleucine	129.08	$9.10e^{-05}$	3.15	1.79	down
Com_14492_pos	Chrysin	254.06	$1.98e^{-05}$	4.29	0.34	up
Com_6405_pos	2-Aminopimelic acid	175.08	$5.55e^{-05}$	2.69	0.58	up
Com_847_pos	Hexadecanedioic acid	268.21	0.0001	3.07	2.10	up
Com_6816_pos	p-Mulegone	154.14	0.0002	4.25	0.40	up
Com_2790_pos	Ethyl oleate	310.29	0.0003	2.02	0.26	up
Com_340_pos	10-Nitrolinoleate	307.21	0.0003	3.23	0.59	up
Com_1373_pos	4-Methyl-5-thiazoleethanol	143.04	0.0005	3.42	0.53	up
Com_7074_pos	4-Methoxycinnamic acid	178.06	0.0007	1.75	1.72	down
Com_790_pos	Avocadyne 1-acetate	308.23	0.0008	1.49	0.64	up
Com_1631_pos	N-[2-chloro-6-(trifluoromethoxy)phenyl]-2,2-dimethylpropanamide	295.06	0.0010	1.30	0.35	up
Com_988_pos	4-Hydroxyisoleucine	147.09	0.0017	2.63	1.69	down
Com_2904_pos	4-(4-nitrophenylazo)aniline	264.06	0.0020	1.34	1.84	down
Com_7463_pos	Polypodine B	496.30	0.0021	2.33	0.73	up
Com_7115_pos	Pyridoxamine	168.09	0.0044	2.92	0.55	up
Com_21629_pos	Xanthurenic acid O-hexoside	367.09	0.0045	2.33	1.49	down
Com_4433_pos	Sedanolide	176.12	0.0055	1.37	0.76	up
Com_3275_pos	11-Deoxy prostaglandin F1α	324.29	0.0056	1.04	0.73	up
Com_8080_pos	Sclareol Glycol	254.22	0.0058	2.20	0.73	up
Com_3186_pos	Prohydrojasmon	254.19	0.0068	1.75	0.58	up
Com_1901_pos	(11E,15Z)-9,10,13-trihydroxyoctadeca-11,15-dienoic acid	310.21	0.0075	1.75	0.66	down
Com_2416_pos	Senkyunolide A	192.11	0.0076	2.89	0.64	up
Com_5858_pos	Apigenin	270.05	0.0081	2.27	0.65	up
Com_3679_pos	Naringenin	272.07	0.0119	1.83	0.64	up
Com_5012_pos	Linalool	154.14	0.0122	2.06	0.68	up
Com_5259_pos	Peimisine	427.31	0.0138	2.51	0.59	up
Com_8057_pos	2-[5-(2-hydroxypropyl)oxolan-2-yl]propanoic acid	184.11	0.0140	1.43	0.73	up
Com_12749_pos	Sclareolide	250.19	0.0154	1.59	1.27	up
Com_9543_pos	Desthiobiotin	214.13	0.0160	2.99	0.33	up
Com_4966_pos	N-Acetyl-L-leucine	173.10	0.0164	1.34	1.38	down
Com_80_pos	Choline	103.10	0.0169	1.24	0.44	up
Com_14222_pos	1-Methyladenine	149.07	0.0169	2.88	1.45	down
Com_14410_pos	Polygalic acid	488.31	0.0170	2.48	1.64	up
Com_8735_pos	L-Saccharopine	276.13	0.0185	1.61	0.71	up
Com_7980_pos	YMK	462.20	0.0212	2.73	3.47	down
Com_9316_pos	Talatisamine	421.28	0.0211	1.10	1.45	down

Com_12428_pos	Oryzalexin E	304.24	0.2190	2.51	1.73	down
Com_1009_pos	4-Methoxybenzaldehyde	136.05	0.0268	1.75	0.73	up
Com_13667_pos	O-p-coumaroyl-O-salicyloyl quinic acid	458.12	0.0276	2.55	1.37	down
Com_18179_pos	myricetin 3-O-beta-D-galactopyranoside	221.07	0.0291	1.23	1.34	down
Com_9674_pos	GKK	309.24	0.0292	1.19	1.29	down
Com_8046_pos	Irganox 259	676.42	0.0303	1.20	0.71	up
Com_14690_pos	6,7-dihydro-5H-dibenzo[d,f][1,3]diazepin-6-one	210.08	0.0343	2.15	0.70	up
Com_4121_pos	N-Acetylneuraminic acid	309.10	0.0372	1.09	1.47	down
Com_1700_pos	Pseudoginsenoside-RT5	654.43	0.0374	1.90	0.52	up
Com_2451_pos	Sunitinib	420.19	0.0400	2.14	3.61	down
Com_4245_pos	Sorbic acid	112.05	0.0412	1.50	0.83	up
Com_9171_pos	6-[2-(2H-1,3-benzodioxol-5-yl)ethyl]-4-methoxy-2H-pyran-2-one	296.07	0.0426	1.07	1.60	down
Com_15122_pos	Moslosooflavone	298.08	0.0428	1.19	1.50	down
Com_1688_pos	Glycerophospho-N-palmitoyl ethanolamine	453.28	0.0435	1.77	0.53	up
Com_1233_pos	N6-Me-dA	265.11	0.0457	1.60	1.41	down
Com_1330_pos	LPE 16:1	451.27	0.0467	1.29	4.21	up
**Negative ion mode**						
Com_6752_neg	N-acetylglycine	117.04	$2.93e^{-06}$	3.46	2.14	down
Com_278_neg	Lumichrome	242.08	$6.14e^{-06}$	2.94	6.07	down
Com_197_neg	7-Methylxanthine	166.05	$1.04e^{-05}$	1.08	1.41	down
Com_982_neg	Prim-O-glucosylcimifugin	468.16	$1.58e^{-05}$	2.42	5.14	down
Com_292_neg	Tetradecanedioic acid	258.18	$2.08e^{-05}$	1.57	2.51	down
Com_14811_neg	p-Coumaric acid ethyl ester	192.08	$3.75e^{-05}$	2.99	0.33	down
Com_10099_neg	8-iso Prostaglandin F2α Ethanolamide	397.28	0.0001	1.93	2.31	down
Com_2695_neg	Danshensu	198.05	0.0002	2.42	2.09	down
Com_7274_neg	DL-α-Methoxyphenylacetic acid	166.06	0.0002	1.38	1.46	down
Com_6206_neg	p-Nitroaniline	138.04	0.0005	1.16	1.46	up
Com_8178_neg	2'-Deoxyadenosine	251.10	0.0007	1.19	0.48	down
Com_299_neg	Nodakenetin	246.09	0.0012	1.26	0.54	down
Com_2236_neg	(±)9(10)-DiHOME	350.22	0.0027	1.36	0.77	up
Com_58_neg	Sinapyl Alcohol	210.09	0.0036	2.04	6.32	up
Com_3060_neg	Arachidonic acid	304.24	0.0041	3.04	0.32	up
Com_3307_neg	Aucubin	346.13	0.0064	2.92	0.40	up
Com_4698_neg	(20R)Ginsenoside Rh2	622.45	0.0064	2.00	0.64	up
Com_575_neg	2-Isopropylmalic acid	176.07	0.0068	2.70	0.60	up
Com_848_neg	Sorbitan monooleate	428.31	0.0072	2.28	0.63	up
Com_6356_neg	Brassinolide	480.35	0.0084	1.92	0.68	up

Com_2406_neg	N-Acetyl-D-alloisoleucine	173.11	0.0137	1.44	1.41	down
Com_1804_neg	Naringerin	272.07	0.0140	1.62	0.68	up
Com_3771_neg	Porphobilinogen	226.10	0.0156	1.98	0.76	up
Com_21728_neg	Ethyl-3-[(1-benzyl-4-piperidyl)amino]-2-cyanoacrylate	313.18	0.0164	2.31	1.29	down
Com_951_neg	LPE 18:2	477.29	0.0177	1.52	1.56	down
Com_8035_neg	Prostaglandin E1 Ethanolamide	443.29	0.0184	1.51	1.47	down
Com_17995_neg	Thiamine	264.10	0.0196	2.28	0.59	up
Com_872_neg	LPE 18:1	479.30	0.0197	1.77	2.35	down
Com_1514_neg	p-Anisaldehyde	136.05	0.0210	2.87	2.79	down
Com_1754_neg	Ascorbic acid	176.03	0.0234	1.26	0.73	down
Com_7912_neg	Cantharidin	196.07	0.0250	1.29	1.85	down
Com_5759_neg	Genipin	208.07	0.0250	2.00	1.45	down
Com_2031_neg	MGMG (18:2)	516.33	0.0347	2.75	0.71	up
Com_2134_neg	13,14-Dihydro prostaglandin E1	356.26	0.0370	1.26	0.75	up
Com_1367_neg	Polygalaxanthone VI	494.14	0.0412	1.92	1.27	down
Com_3420_neg	L-Ornithine	132.09	0.0424	1.08	0.62	up
Com_1647_neg	Oxyresveratrol	244.07	0.0425	1.04	0.68	up
Com_13223_neg	3,5-Dimethoxy-4-hydroxybenzaldehyde	182.06	0.0427	1.50	1.25	down
Com_2821_neg	C-hexosyl-luteolin O-p-coumaroylhexoside	756.19	0.0483	1.27	0.56	up
Com_3792_neg	β-Estradiol	544.36	0.0498	2.88	0.72	up
